# A Potential Role of CD82/KAI1 during Uterine Decidualization in Mice

**DOI:** 10.3390/cimb46030118

**Published:** 2024-02-27

**Authors:** Qijun Li, Mengyao Song, Ke Cao, Qian Zhang

**Affiliations:** 1 Laboratory Animal Center, Chongqing Medical University, Chongqing 400016, China; liqijun9889@163.com (Q.L.); mengyao_s@163.com (M.S.); caoke9005@163.com (K.C.); 2 Chongqing Engineering Research Center for Rodent Laboratory Animals, Chongqing 400016, China

**Keywords:** CD82/KAI1, decidualization, stromal cells, uterus, mouse

## Abstract

The tumor metastasis suppressor gene CD82/KAI1 has been demonstrated to impact human trophoblast invasion and migration. Communication between trophoblasts and decidual stromal cells plays a crucial role in controlling the normal invasiveness of trophoblasts. However, whether CD82/KAI1 is involved in decidualization and what role it plays remain unclear. CD82/KAI1 demonstrates specific spatiotemporal expression patterns in stromal cells undergoing decidualization during pregnancy. This is observed in both naturally pregnant females post-implantation and pseudopregnant mice undergoing induced decidualization, as detected through in situ hybridization and immunofluorescence. CD82/KAI1 expression showed a significant time-dependent increase in cultured stromal cells after 24 and 48 h of progesterone (P_4_) and estrogen (E_2_) treatment. This was accompanied by a notable upregulation of decidualization markers, including cyclin D3 and PR. After transducing stromal cells with the adenovirus-overexpressing CD82/KAI1 for 48 h, the expression of cyclin D3 protein increased. Meanwhile, there was an attenuated expression of CD82/KAI1 due to an adenovirus siRNA knockdown, whereas cyclin D3 and PR expressions were not affected. Our findings suggest a potential role of CD82/KAI1 in regulating the process of decidualization, providing insights into stromal cell differentiation.

## 1. Introduction

In early pregnancy, the process of decidualization involves spindle-shaped fibroblasts gradually enlarging and rounding under the influence of steroid hormones [1,2,3]. Trophoblast cells come into contact with the decidualized endometrium, and the maternal–fetal interface gradually forms, playing a role in the material exchange and nutrient supply to maintain a normal pregnancy [4,5,6]. Decidualization plays a critical role in successful implantation and preventing early embryo miscarriage [7]. Understanding this process thoroughly is essential for enhancing the chances of successful births.

CD82/KAI1 belongs to the transmembrane 4 superfamily and is considered a critically important tumor metastasis suppressor [8,9]. Deletion of the CD82/KAI1 gene results in increased tumor metastasis in various tissue types, such as the liver, colon, prostate, lungs, bladder, breasts, and more [10]. Overexpression of CD82/KAI1 transduced into oral cancer cell lines can significantly inhibit cell invasion [11]. CD82/KAI1 demonstrates a widespread inhibitory effect on cell migration and invasion. In our earlier investigation, we noticed a regular expression of CD82/KAI1 in the human uterus. CD82/KAI1 is also expressed in human trophoblast cells, exerting a significant impact on MMP9 activity. In the early stages of pregnancy, CD82/KAI1 plays a crucial negative regulatory role at the maternal–fetal interface by effectively suppressing the invasion and migration of human trophoblast cells [12]. Cross-talk between trophoblast and decidual stromal cells controls the physiological balance of trophoblast invasiveness in paracrine and autocrine ways [13]; it remains unclear whether CD82/KAI1 functions during the process of decidualization. Therefore, we have initiated this research.

Decidualization occurs only in species with forms of hemochorial placentation, in which the trophoblast invades the uterus, such as in humans, rats, mice, and nonhuman primates [14]. Decidualization in humans is similar to that in mice. Mouse uterine decidualization begins around day five (D5, the presence of vaginal plugs recorded as day one) of pregnancy after embryo implantation, which involves the proliferation, differentiation, and polyploidy of endometrial stromal cells [1]. Increasing the levels of progesterone (P_4_) and preimplantation estrogen on D4 promotes stromal cell proliferation, creating an optimal environment for blastocyst attachment [15,16]. Subsequently, the primary decidual zone (pdz) forms during D5–D6, followed by stromal cells around the implanting embryo ceasing to proliferate and undergoing differentiation. Once apoptotic in the pdz, cells persistently proliferate and differentiate outside the pdz. This process leads to the formation of the secondary decidual zone (sdz) on D7–D8. In order to accommodate embryonic growth, cells in the sdz eventually undergo apoptosis [2,17]. Therefore, we employed in situ hybridization, Western blotting, and immunofluorescence to examine the expression of CD82/KAI1 in a mouse uterus. In addition, we also used adenovirus to overexpress and adenovirus-delivered siRNA to silence CD82/KAI1 in cultured stromal cells to explore its regulatory function.

## 2. Materials and Methods

### 2.1. Animals and Treatments

CD-1 mice at the age of 6–8 weeks were obtained from Chongqing Medical University (SCXK 2022-0010). The experimental procedures involving mice in this study received approval from the Animal Care and Use Committee of Chongqing Medical University (IACUC-CQMU-2021064).

Mating pairs were established by pairing female mice with fertile, normal male mice or by pairing female mice with males in which the vas deferens had been surgically removed. And presence of vaginal plugs was used to designate day 1 (D1) of pregnancy or pseudopregnancy. Mice were euthanized through intraperitoneal injection of 150 mg/kg sodium pentobarbital (P3761, Sigma, St. Louis, MO, USA), followed by cervical dislocation. Subsequently, uteri were collected at various stages of pregnancy for subsequent analysis. The presence of embryos in the reproductive tracts confirms pregnancy on D1–D4. For D5–D6 pregnant mice, tail vein injection of 1% trypan blue was performed to identify the implantation site. Implantation sites for D7–D8 pregnant mice were determined by observing embryo morphology. Tissue samples were gathered and preserved at −80 °C for subsequent analysis using in situ hybridization and Western blotting. Additionally, some were fixed in 4% paraformaldehyde (PFA) at 4 °C for immunohistochemical analysis.

To induce artificial decidualization in the uteri of mice during pseudopregnancy, 10 µL of sesame oil (S3547, Sigma, Steinheim, Germany) was administered by injection into one uterine horn on D4. The contralateral uninjected horn was used to control (Supplemental Figure S1). Pseudopregnancy mice were sacrificed between D5 and D8 to collect the uteri. The presence of decidual cell reaction was verified through an elevation in uterine weight and histological examination.

### 2.2. In Vitro Isolation and Decidualization Induction of Uterine Stromal Cells

Isolation of primary endometrial stromal cells from mice was performed following established procedures as previously outlined [18]. In brief, uterine horns were dissected to eliminate adipose tissue, followed by longitudinal incisions and sectioning of the uteri into 2–3 mm fragments from D4 pregnant mice. The fragments of the uterine tissue were placed in a sterile petri dish. Subsequently, these tissue sections underwent a series of washing steps with phosphate-buffered saline (PBS) and were sequentially treated with 0.25% trypsin (27250018, Invitrogen, Carlsbad, CA, USA) for 1 h at 4 °C, followed by 1 h of treatment at 37 °C. After the digestion process, these tissues were diluted in PBS containing 10% charcoal-stripped fetal bovine serum (FBS; F6765, Sigma, St. Louis, MO, USA), and were gently mixed by pipetting repeatedly (over 50 times) to remove epithelial clumps. In the subsequent steps, the remaining tissues were subjected to two washes in PBS. After being washed, the tissues were immersed in PBS containing 0.5% Type II collagenase (17101015, Invitrogen, Carlsbad, CA, USA) at 37 °C for 30 min. Subsequently, these tissues were placed into PBS containing 10% FBS to terminate digestion. After two rinses with PBS, the tissues were subjected to repetitive pipetting (over 50 times) using a 1 mL pipette to ensure thorough mixing. The process was continued until the liquid became turbid due to the presence of dispersed stromal cells, at which point it was terminated. The cells underwent purification via a 70 μm nylon filter and subsequent centrifugation. Then, the cells were subjected to two washes using fresh phenol-red-free culture medium (1:1, *v*/*v*; DMEM/Ham’s F-12; 11039021, Invitrogen, Carlsbad, CA, USA). The cells were subsequently seeded at a density of 2 × 10^5^ cells per 6-well cell culture plate for primary culture. One hour later, after removing unattached cells through multiple rinses with fresh cell culture medium, the culture was sustained in fresh cell culture medium. This medium was further supplemented with 10 nM estradiol (E_2_; E2257, Sigma, St. Louis, MO, USA) and 1 μM progesterone (P_4_; P6149, Sigma, St. Louis, MO, USA). These conditions were maintained in a 37 °C, 5% CO_2_ incubator.

### 2.3. In Situ Hybridization (ISH) 

The method of ISH was used as published before [19]. ISH was performed using digoxigenin (DIG)-labeled probes. The DIG labeling process followed the manufacturer’s instructions (Roche Diagnostics, Mannheim, Germany). The tissue sections were subjected to fixation in 4% PFA for 1 h, followed by three consecutive 5 min washes with PBS. Subsequently, the sections underwent a 20 min incubation with 1% Triton-X100, followed by an additional three rounds of 5 min PBS washes. After pre-hybridization, digoxigenin (DIG)-labeled probes were allowed to hybridize overnight at 50 °C. The hybridization buffer comprised 200 mM NaCl, 13 mM Tris, 5 mM sodium phosphate monobasic, 5 mM sodium phosphate dibasic, 5 mM EDTA, 50% formamide (*v*/*v*), 10% dextran sulfate sodium (DSS, *w*/*v*), 1 mg/mL salmon sperm DNA, 2% bovine serum albumin (BSA, *w*/*v*), and a DIG-labeled probe (final dilution of 1:200 obtained from the reaction with 1 μg template DNA). After performing two post-hybridization washes (1× SSC, 50% formamide, 0.1% tween-20) at 55 °C, two buffer I (150 mM NaCl, 100 mM Tris, pH 7.5) washes were then performed. Sections were incubated with 1:2000 diluted anti-DIG antibody (Roche) overnight after being blocked by 2% blocking reagent for 1 h. Slides were washed in buffer II (100 mM NaCl, 50 mM MgCl_2_, 100 mM Tris, pH 9.5) following four 20 min washes in buffer I. Nitro blue tetrazolium/5-bromo-4-chloro-3-indolyl-phosphate (NBT/BCIP) was then incubated in accordance with the manufacturer’s instructions (Promega, Madison, MI, USA). The slides underwent counterstaining using nuclear fast red, followed by dehydration and clearing in xylene, before being mounted in neutral resin.

### 2.4. Immunofluorescence (IF) and Immunocytochemistry (ICC)

The uterine tissues were preserved by fixation in 4% PFA and then paraffin-embedded. They were then cut into 5 μm sections, dewaxed in xylene, and rehydrated through an ethanol gradient. Normal goat serum was utilized for section blocking. The primary antibody used was rabbit polyclonal CD82/KAI1 (ab66400, Abcam, Cambridge, UK) at a dilution of 1:200, followed by the secondary antibody fluorescein isothiocyanate (FITC)-conjugated anti-rabbit IgG (ZB-0312, Zhongshan Biotechnology, Zhongshan, China) at a 1:200 dilution. Propidium iodide (PI; 5 μg/mL; 537059, Sigma, San Diego, CA, USA) was employed for nuclear staining.

Fix stromal cells in 4% PFA for 20 min, permeabilize with 0.25% Triton X-100 for 10 min, and subsequently block with 1% bovine serum albumin (BSA) for 1 h. Employ an indirect immunolabeling approach using mouse anti-vimentin (1:500; ab20346, Abcam, Cambridge, UK) or mouse anti-cytokeratin (1:300; ab6401, Abcam, Cambridge, UK) as the primary antibodies. Subsequently, the secondary antibody (FITC) diluted at 1:200 was used for 1 h and counterstained with PI (5 μg/mL) for 10 min.

### 2.5. Western Blotting

Proteins were extracted from various stages of treated uterine tissues or cells using a whole-cell lysis buffer containing an inhibitor cocktail. The measurement of proteins was carried out with the BCA Protein Assay Kit (23225, Thermo Scientific, Waltham, MA, USA). SDS-polyacrylamide gel electrophoresis was employed to separate 20 μg of protein extracts. Subsequently, polyvinylidene difluoride membranes were employed for the transfer of the separated proteins. After blocking for 1 h at 25 °C with 5% TBST, the membranes were subjected to an overnight incubation at 4 °C in the presence of the following primary antibodies (rabbit anti-CD82/KAI1 (1:500, ab66400, Abcam), mouse anti-cyclin D3 (1:1000, 2936, Cell Signaling Technology, Danvers, MA, USA), rabbit anti-progesterone receptor (anti-PR, 1:500, SC-538, Santa Cruz, Santa Cruz, CA, USA) and rabbit anti-β-actin (1:2000, 4970, Cell Signaling Technology, Danvers, MA, USA)). The membranes underwent a 1-h incubation at room temperature with monoclonal secondary antibodies that were conjugated with HRP following three washes in TBST. Enhanced chemiluminescence (Pierce Chemical, Rockford, IL, USA) was used to detect immunoreactive bands.

### 2.6. Semiquantitative RT-PCR

Extraction of total RNA from stromal cells after decidualization induction using Trizol reagent (15596026, Invitrogen, Waltham, MA, USA) following the manufacturer’s guidelines. Subsequently, the obtained 2 μg RNA was reverse transcribed into cDNA utilizing a reverse transcription kit (4387406, Invitrogen, Foster City, CA, USA), according to the instructions of the manufacturer. PCR was performed in a 25 µL reaction volume, with the number of cycles ranging from 22 to 30, determined based on the amplification of the target gene. The mouse housekeeping gene actin served as an internal control. Specific primers are provided in Supplemental Table S1.

### 2.7. CD82/KAI1 REcombinant Adenovirus Purification and Infection 

CD82/KAI1 recombinant adenovirus and adenovirus-delivered siRNA (obtained from Professor Tong-Chuan He, University of Chicago Medical Center, Chicago, IL, USA) were expanded to infect HEK293 cells as previously described [20]. When most cells emitted red fluorescent, the cells were collected, repeatedly frozen, and thawed 3–4 times, and then centrifuged to obtain recombinant adenovirus supernatant. Finally, a large amount of virus supernatant was obtained, which was centrifuged and purified by AdEasy Virus Purification Kits (240243, Agilent, Santa Clara, CA, USA). 

Virus particle titer was determined by spectrophotometry, and the concentration of the titer was subsequently modified to reach a level of 10^11^ PFU/mL. The stromal cells were isolated and incubated in 10% FBS medium containing 2 μL adenovirus supernatant (10^11^ PFU/mL) into 6-well cell culture plate for 48 h. After end of the culture, the cells were collected for Western blotting.

CD82/KAI1siRNA sense sequence (5′-GCGAGAAGATCAAGGAAGA-3′) and antisense sequence (5′-TCTTCCTTGATCTTCTCGC-3′).

### 2.8. Statistical Methods

Western blotting was quantified by MetaView Image Analyzing System (Version 4.50, Universal Imaging Corp., Downingtown, PA, USA). Results were presented as the mean ± standard deviation. Significance among groups was evaluated using *t*-test and one-way analysis of variance (one-way ANOVA). A significance level of *p* < 0.05 was considered statistically significant. **, *p* < 0.01; *, *p* < 0.05.

## 3. Results

### 3.1. Expression of CD82/KAI1 in Mouse Uteri during Pregnancy

**Figure 1:**
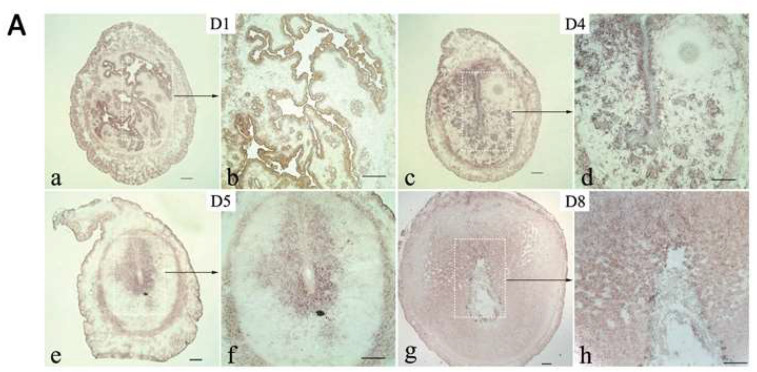

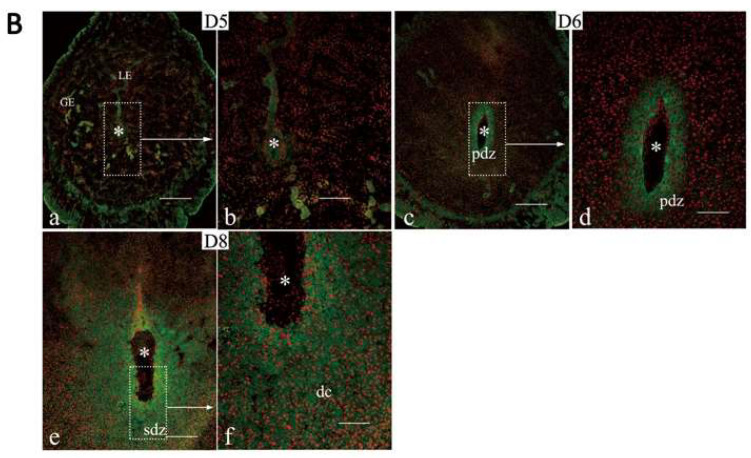
Expression of CD82/KAI1 during early pregnancy. (**A**) In situ hybridization of CD82/KAI1 in mouse uterus on D1, D4, D5 and D8 of pregnancy. The positive signal was visualized as a dark-brown color. Scale bars in (**a**,**c**,**e**,**g**): 500 μm; scale bars in (**b**,**d**,**f**,**h**): 200 μm. (**B**) CD82/KAI1 immunofluorescence in mouse uteri during pregnancy on D5, D6 and D8. FITC-labeled CD82/KAI1 antibody in green, propidium iodide (PI) labeled nuclei in red. Scale bars in (**a**,**c**,**e**): 500 μm; scale bars in (**b**,**d**,**f**): 200 μm; pdz, primary decidual zone; sdz, secondary decidual zone; *, embryo; GE, glandular epithelium; LE, luminal epithelium; dc, decidual cells.

We initially utilized in situ hybridization (ISH) to investigate the mRNA expression pattern of CD82/KAI1 in the mouse uterus at various phases of the pregnancy. CD82/KAI1 was observed in uterine luminal epithelial cells on D1 (Figure 1A(a,b)), and the expression extended to glandular epithelial cells on D4 (Figure 1A(c,d)). On the morning of D5, following the initiation of embryo attachment, there was a notable increase in the specific expression of CD82/KAI1 observed around the site of the embryo implantation (Figure 1A(e,f)). During the decidualization process in the uterus on D8 (Figure 1A(g,h)), this expression pattern was further extended to the primary and secondary decidual zones. CD82/KAI1 immunofluorescence was conducted, focusing on the period from D5 to D8 to clarify the protein pattern during this decidualization. The findings demonstrated a uniform expression pattern consistent with ISH. On D5, CD82/KAI1 was positioned in the cells surrounding the implantation site (Figure 1B(a,b)). From D6 to D8, CD82/KAI1 expression was widespread in the pdz (Figure 1B(c–f)). Particularly, a strong expression was observed in the sdz on D8 (Figure 1B(e,f)). The consistent presence of CD82/KAI1 was observed in both the myometrium and serosa across the majority of examined samples, in addition to the described expression pattern (Figure 1B). Meanwhile, the expression of CD82/KAI1 increased from D1 to D6, with significantly higher levels observed at the inter-implantation sites on D5 and D6 compared to the implantation sites based on Western blotting (Supplemental Figure S2). The expression of CD82/KAI1 in the uterus during the initial phases of pregnancy (D1 to D6) provides evidence supporting its role in stromal cell decidualization.

### 3.2. Expression of CD82/KAI1 in Mouse Uterus during Artificially Induced Decidualization

**Figure 2:**
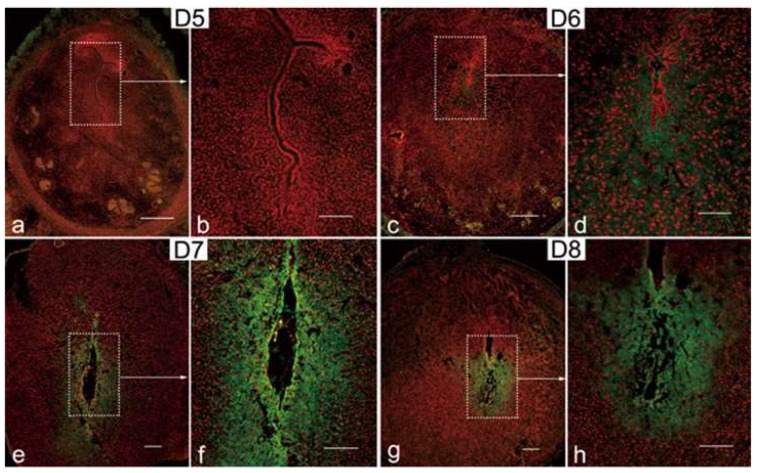
Expression of CD82/KAI1 during artificially induced decidualization. (**a**–**h**) CD82/KAI1 immunofluorescence detection in murine pseudopregnant uteri undergoes oil-induced artificial decidualization on D5 (**a**,**b**), D6 (**c**,**d**), D7 (**e**,**f**) and D8 (**g**,**h**), showing a similar expression pattern compared with normal pregnancy. FITC-labeled CD82/KAI1 antibody in green, propidium iodide (PI)-labeled nuclei in red. Scale bars in (**a**,**c,e**,**g**): 500 μm; scale bars in (**b**,**d**,**f**,**h**): 200 μm. Similar results were obtained in two to three independent mice.

To verify whether the expression of CD82/KAI1 is triggered by the uterine decidualization process or contingent upon the existence of a viable embryo, we examined the expression of CD82/KAI1 in a model where uterine decidualization was artificially induced through intraluminal oil infusion. In Figure 2, the signals of CD82/KAI1 became evident in stromal cells on D5, showing a concentrated localization, particularly around the implantation attachment site (Figure 2a,b). From D6 to D8, there was an increasing trend in pdz expression that was specific to the region (Figure 2c–h). The location signal of CD82/KAI1 closely resembles that observed during normal implantation and decidualization, indicating that CD82/KAI1 expression is primarily dependent on the uterine decidualization process.

### 3.3. Expression of CD82/KAI1 Increases during In Vitro Decidualization of Stromal Cells

**Figure 3:**
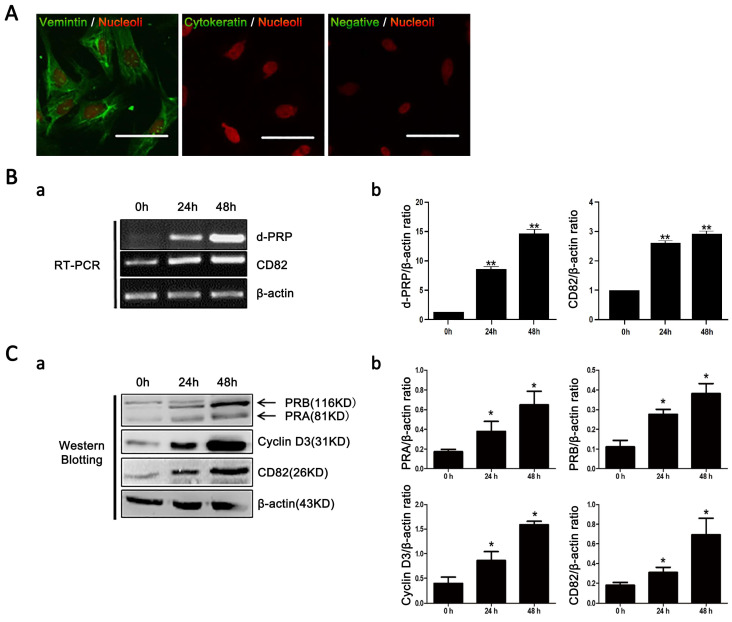
Mouse uterine stromal cells undergo decidualization in primary culture. (**A**) Immunofluorescence analysis of vimentin and cytokeratin staining in cultured stromal cells. The vimentin positive, cytokeratin-negative staining demonstrated that these cells are of stromal origin. (**B**) (**a**) RT-PCR analysis to dPRP and CD82/KAI1 mRNA expression in cultured stromal cells under progesterone (P_4_) and estrogen (E_2_) treatment up to 48 h to indicated decidualization. (**b**) Densitometric values from RT-PCR analysis in stromal cells cultured up to 48 h (mean ± SE of three independent experiments, *t*-test; ** *p* < 0.01). (**C**) (**a**) Western blotting analysis of CD82/KAI1, PRA, PRB and cyclin D3 proteins in cultured stromal cells under progesterone (P_4_) and estrogen (E_2_) treatment up to 48 h to indicated decidualization. (**b**) Densitometric values from Western blotting analysis in stromal cells cultured up to 48 h (mean ± SE of three independent experiments, *t*-test; * *p* < 0.05).

To delve deeper into the correlation between CD82/KAI1 expression and the decidualization process in uterine cells, we established an in vitro model of primary mouse stromal cell decidualization. We monitored the purity of isolated uterine stromal cells by performing immunofluorescence staining. To ensure low contamination of isolated uterine stromal cells with epithelial cells, we identified vimentin-positive and cytokeratin-negative cells, as shown in Figure 3A. After 24–48 h of P_4_ and E_2_ treatment, we analyzed the expression of decidualization marker genes, including PR (protein *p* < 0.05; Figure 3C(a)), Cyclin D3 (protein *p* < 0.05; Figure 3C(a)), and a decidual prolactin-related protein (dPRP, mRNA *p* < 0.05; Figure 3B) by using Western blotting and RT-PCR [21]. The results demonstrated a consistent elevation of gene expression in stromal cells undergoing decidualization in culture. In this context, it was noted that the expression of CD82/KAI1 gradually rose at both the mRNA (*p* < 0.05; Figure 3B) and protein (*p* < 0.05; Figure 3C(b)) levels, with decidualization progression in stromal culture cells. These findings reveal the potential role of CD82/KAI1 in the process of stromal cell decidualization.

### 3.4. CD82/KAI1 Plays a Role in Stromal Cell Decidualization

**Figure 4:**
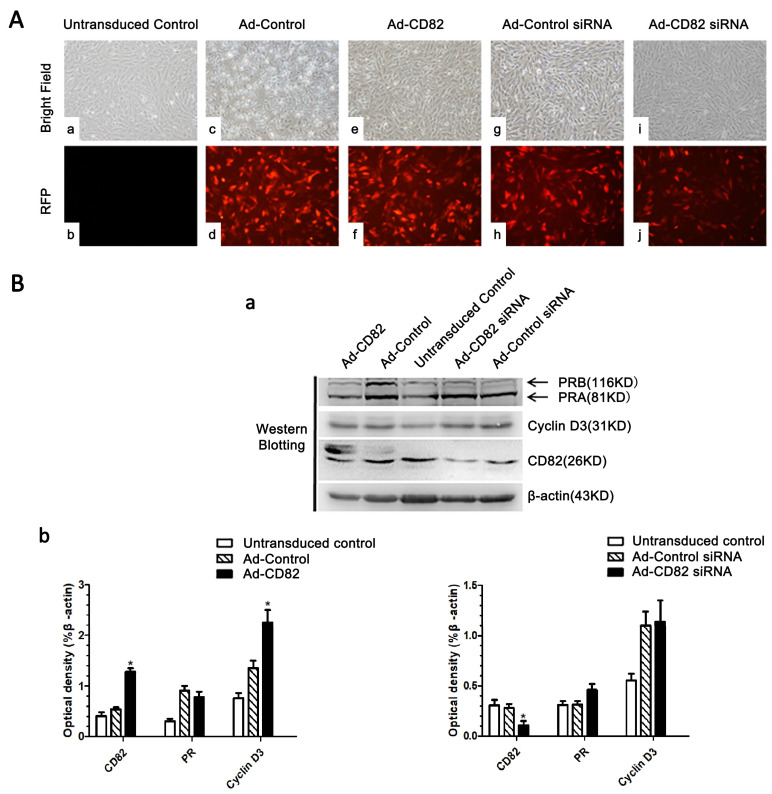
Adenovirus to overexpress and adenovirus-delivered siRNA to silence of CD82/KAl1 in cultured stromal cells. (**A**) Stromal cells (**c**,**e**,**g**,**i**) transduced with adenovirus, showing the transduction efficiency. Stromal cells (**a**) as a control transduced nothing. Stromal cells (**a,c,e,g,i**) under the light microscope and stromal cells (**b,d,f,h,j**) the fluorescence microscope (**a**–**j** at 100× magnification). (**B**) (**a**) Western blotting analysis of CD82/KAI1, PRA, PRB and cyclin D3 proteins in the stromal cells after transducing treatment cultured to 48 h. (**b**) Densitometric values from Western blotting analysis of CD82/KAI1, PRA, PRB and cyclin D3 proteins (mean ± SE of three independent experiments, *t*-test; * *p* < 0.05 as compared with control).

To evaluate the impact of CD82/KAI1 on stromal cell decidualization, we employed adenovirus to overexpress and adenovirus-delivered siRNA to silence CD82/KAI1 in cultured stromal cells. Successful transduction was confirmed by observing red fluorescence after 48 h (Figure 4A). The Western blotting results show the overexpression of the CD82/KAI1 adenovirus, which markedly elevated the levels of the CD82/KAI1 protein (*p* < 0.05; Figure 4B). Moreover, this CD82/KAI1 overexpression resulted in an upregulation of cyclin D3 protein expression in decidual stromal cells (*p* < 0.05; Figure 4B). Meanwhile, transduction with CD82/KAI1 adenovirus-delivered siRNA reduced the expression level of the CD82/KAI1 protein (*p* < 0.05; Figure 4B). However, the expression levels of cyclin D3 and PR were not affected. These findings imply that CD82/KAI1 overexpression upregulates cyclin D3 expression, and CD82/KAI1 plays a regulatory role in stromal cell decidualization.

## 4. Discussion

The normal decidualization process of uterine stromal cells plays a crucial role in facilitating the intricate process of successful embryo implantation within the endometrium [22]. The expression of CD82/KAI1 is consistently observed throughout the menstrual cycle in human endometrium and the maternal–fetal interface. Notably, CD82/KAI1 was localized to decidual cells, with its pronounced expression particularly evident in samples from the first and second trimesters, corresponding to a higher proportion of decidual tissue during these stages [23]. The decidual process exhibited similarities between humans and mice. In our earlier studies, we found that CD82/KAI1 exhibited spatiotemporal expression during pregnancy. Before embryo implantation, CD82/KAI1 was expressed in the luminal epithelial cells from D1 to D4. In mice, embryo implantation was initiated around D5 of pregnancy. Stromal cells surrounding the implanting embryo underwent a decidual transformation, marked by extensive proliferation and differentiation into specialized cell types with polyploidy [1]. CD82/KAI1 was specifically expressed around the implantation site from D5 to D8. As our study focused on the decidualization process, we employed immunofluorescence to monitor the dynamic changes in CD82/KAI1 from D5 to D8. Our observations reveal that CD82/KAI1 participated in the process of stromal cell decidualization. Parallel findings by Qu et al. [24] in human tissues suggest that CD82/KAI1 could serve as a novel promoter for decidualization within a positive feedback loop. In other words, CD82/KAI1 is found in stromal cells when decidualization is induced artificially using oil, revealing that the expression of CD82/KAI1 is not straightforwardly dependent on the implantation of blastocysts. The role played by CD82/KAI1 in this intricate process needs further in-depth research and exploration.

Cyclin D3 has been demonstrated to play a crucial role in mouse decidualization [17]. In the early stages of decidualization (D5–D7), there was an increase in the expression of cyclin D3 mRNA in stromal cells located at both the mesometrial and antimesometrial poles. By D8, the signals were mainly concentrated in stromal cells within the mesometrial decidual bed. Cyclin D3 exhibited primary expression in stromal cells undergoing decidualization, emphasizing its pivotal role in the intricate process of implantation [25,26]. The adenovirus overexpressed CD82/KAI1, which significantly upregulated the expression of cyclin D3 protein in stromal cell transduction for 48 h. Meanwhile, silencing CD82/KAI1 did not reduce the expression levels of cyclin D3 and PR, suggesting potential compensatory mechanisms by other genes within the transmembrane 4 superfamily (TM4SF). As a modulator of cell membrane heterogeneity, CD82/KAI1 alters microdomains, trafficking, and the topography of the membrane by changing the landscape of membrane molecules [27]. This process plays a significant role in cell–cell and cell–ECM interactions during the decidualization of stromal cells [28]. Therefore, CD82/KAI1 may potentially engage in the modulation of cell–cell and cell–matrix interactions in an indirect manner, contributing to stromal cell decidualization. Subsequently, we can proceed to the next step of research by investigating the interaction mechanisms with other transmembrane proteins.

Therefore, our results indicate that CD82/KAI1 plays a crucial role in the decidualization process of endometrial stromal cells following embryo implantation. The findings suggest that CD82/KAI1 potentially regulates maternal–fetal dialogue and exerts an impact on cell cycle dynamics at the maternal–fetal interface during the decidualization process. Further experiments are needed to confirm the precise mechanisms involved.

## Data Availability

All data and material generated or analyzed during this study are included in this published article and Supplementary Materials.

## Supplementary Materials

The following supporting information can be downloaded at: https://www.mdpi.com/article/10.3390/cimb46030118/s1, Figure S1: Induction of artificial decidualization in mice during pseudopregnancy. On Day 4, 10 µL of sesame oil was injected into one uterine horn (+), while the contralateral uninjected horn (−) served as a control; Figure S2: Western blotting analysis of CD82/KAI1 protein expression in the normal pregnancy mice. (A) Western blotting analysis of CD82/KAI1 protein expression in the normal pregnancy mice endometria from D1–D6. (IS: implantation sites, IIS: inter-implantation sites). (B) Densitometric values from western blotting analysis of CD82/KAI1 protein in mouse uterus on D1, D4, D5 and D6 of pregnancy (mean ± SE of three independent experiments, one-way ANOVA; * *p* < 0.05, *** *p* < 0.001); Table S1: Primers used in this study.
