# Supplementary material for: A Potential Role of CD82/KAI1 during Uterine Decidualization in Mice
Source: Curr Issues Mol Biol. 2024 Feb 27;46(3):1799–809. doi: 10.3390/cimb46030118 (PMC10969677; doi:10.3390/cimb46030118)
Supplement: Supplementary file 1 [file cimb-46-00118-s001.zip › cimb-2882116-supplementary.pdf]

Supplemental Material

Supplemental Table S1. Primers used in this study.

Gene Name	Product Size	Melting Temperature	Primers Sequences	NM-Numbers
dPRP	138 bp	58 °C	forward (5′-TTATGG GTGCATGGATCACTCC-3′); reverse (5′-CCCACGTAAGGTCATCATGGAT-3′)	NM_001289919.1
Cd82/Kai1	294 bp	58 °C	forward (5′-ACCACTTACCCATGCTCCTG-3′) ; revers(5′-TCAGTACTTGGGGACCTTGC-3′)	NM_001136055.2
β-actin	228 bp	58 °C	forward (5′-AGCCATGTACGTAGCCATCC-3′); reverse (5′-CTCTCAGCTGTGGTGGTGAA-3′)	NM_007393.5

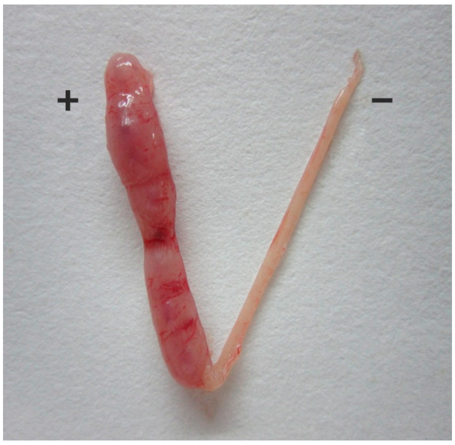

Supplemental Figure S1. Induction of artificial decidualization in mice during pseudopregnancy. On Day 4, 10 μL of sesame oil was injected into one uterine horn (+), while the contralateral uninjected horn (−) served as a control.

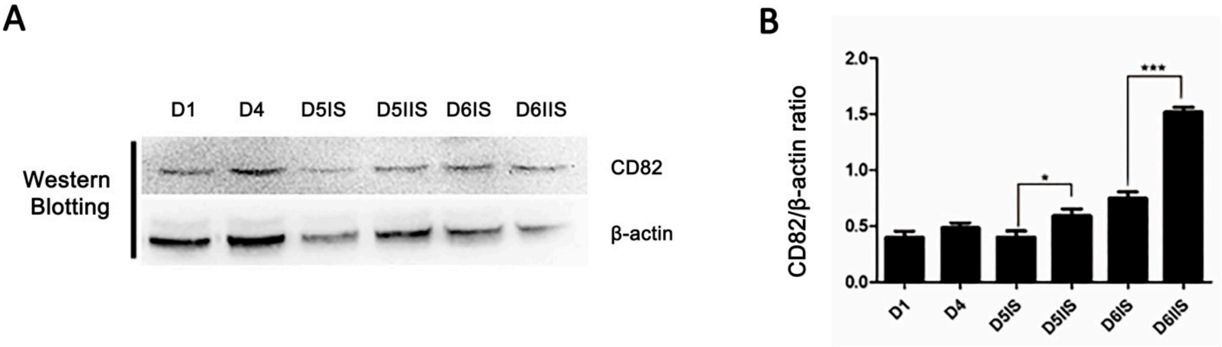

Supplemental Figure S2. Western blotting analysis of CD82/KAI1 protein expression in the normal pregnancy mice. (**A**) Western blotting analysis of CD82/KAI1 protein expression in the normal pregnancy mice endometria from D1-D6. (IS: implantation sites, IIS: inter-implantation sites). (**B**) Densitometric values from western blotting analysis of CD82/KAI1 protein in mouse uterus on D1, D4, D5 and D6 of pregnancy (mean ± SE of three independent experiments, one-way ANOVA; * $p < 0.05$, *** $p < 0.001$).
